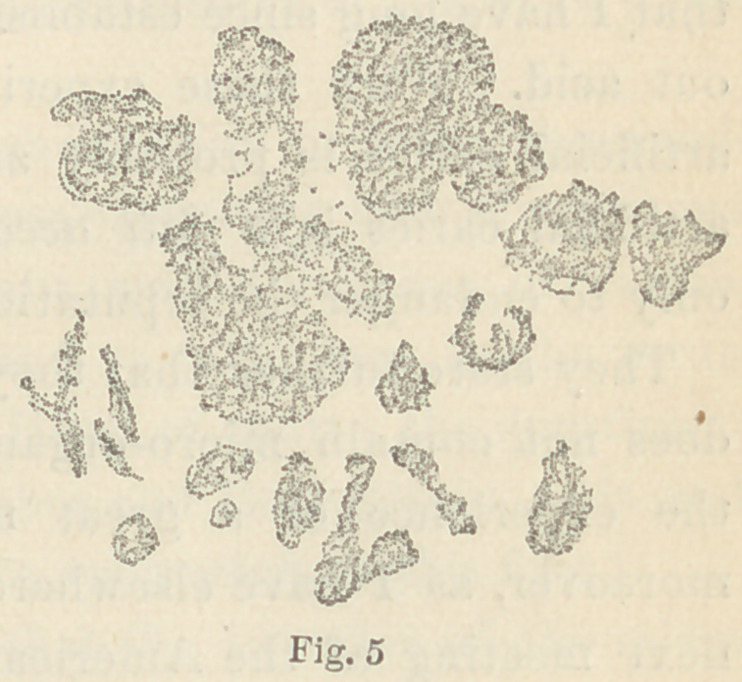# Fermentation in the Human Mouth

**Published:** 1884-07

**Authors:** W. D. Miller

**Affiliations:** Berlin, Germany


THE
### Independent Practitioner.

VOL. V. JULY, 1884. NO. 7.

### Original Communications.

### FERMENTATION IN THE HUMAN MOUTH.

### THE FUNGI OF DENTAL CARIES; THEIR PURE CULTIVATION AND EFFECT UPON LOWER ANIMALS.

BY DR. W. D. MILLER, BERLIN, GERMANY.

(Continued from page 291.)

In the May number of the INDEPENDENT PRACTITIONER will be found the description and illustrations of two species of micro-organisms which I had, up to the first of March, 1884, obtained from carious dentine. These species I isolated by inoculating culture liquids with very small pieces of carious dentine, taken from near the border of the normal tissue. If the fungus was not at once obtained in the pure state, a second culture tube was inoculated after the method of fractional culture, with a minimum portion of the first, and so on.

It soon, however, became apparent that the capture of these two species by no means ended the work; on the other hand, new forms continually presented themselves, and in order to be able to determine definite characteristics for each species, resort was had to the culture on plates of gelatine prepared with beef extract, calf's broth, malt decoction, etc.

The beef-extract gelatine, for example, I prepare as follows: 200 c. c. water + 3,0 beef extract + 3,0 sugar, are first neutralized, then slowly boiled for five minutes, and filtered (filter and all other vessels, of course, sterilized). After cooling, 8,0 of the finest gelatine is added and gradually heated till the gelatine is dissolved ; it is then cleared with the white of an egg, and all together kept at the boiling point for about five minutes, stirring constantly to prevent burning ; it is then passed through a filter, surrounded by a bath of boiling water, into glass tubes with cotton stoppers (both sterilized), and kept in a refrigerator. When to be used it is melted in warm water and poured upon sterilized cold glass plates, which may be m. 0,15 long by m. 0,07 wide, and placed in the moist chamber. The layer of gelatine should be about two m. m. thick.

Suppose now we have a culture containing different species of fungi, and we wish to separate them. A thin platinum wire with one end melted into a glass rod is sterilized in the flame of a bunsen burner, and on cooling dipped into the impure culture and lightly drawn across the surface of the gelatine; the fungi which adhered to the platinum wire are thereby scattered in a row upon the surface of the gelatine, and in a short time we will find that at certain points in the row one form of fungus has developed, and at other points other forms. Now if we take upon the end of our platinum wire a small quantity of fungi from one of these points and draw it across the surface of a second plate, we will, in parts of this line, invariably obtain a pure culture of one of the species in the original impure culture, nearly every species being distinguished by some characteristic in the form which it takes in growing, and in its action upon the gelatine. Having obtained a pure culture in this manner, test-tubes containing gelatine are inoculated with it. In these it may be kept in a pure state for weeks, or months, while the plates are always short-lived.

The gelatine method of pure culture has one great disadvantage in the low melting point of the gelatine. Twenty-four to twenty-five degrees Centigrade is the highest temperature to which they can be exposed without danger of melting, and this, to fungi which are accustomed to a temperature of thirty-seven degrees Centigrade, is not always a matter of indifference. I have succeeded in isolating three species, besides

Fig. 1

the ones described in the May number of this journal, and, for the purpose of distinguishing them only, I will designate them by the Greek letters $\gamma$, $\delta$ and $\varepsilon$. These fungi are shown in Figs. 2, 3 and 4. In Fig. 1 I have reproduced the fungus described on page 226 as a caries fungus, for the sake of comparison. When the species $\alpha$, $\gamma$ and $\delta$ are isolated, it is not difficult to tell one from the other; when, however, they are mixed together, it is next to impossible to determine which is which, and especially is this the case with $\alpha$ and $\gamma$. Their modes of development on gelatine are, however, so different, that we possess therein a ready means of distinguishing between them.

Fig. 2

Fig. 3

The $\alpha$ fungus, *sparingly* inoculated into gelatine tubes, presents in a few days the appearance which I have attempted to represent in Fig. 1$\alpha$. It may be compared to a bunch of grapes, which presents all gradations from the fully developed berry to the little green one; the masses of fungi are globular or ovoid, exceedingly fine, and semi-transparent, presenting altogether a strikingly beautiful culture, which it is impossible to even approximately represent by drawing. It furthermore forms a button upon the surface of the gelatine; the latter becomes softened but not liquefied. On the plates it presents soft, milky ridges or knots, raised sometimes a m. m. above the surface of the gelatine, and obtaining a width at the base of three to six m. m. The $\gamma$ fungus differs from all other fungi that I have yet found in decaying dentine, in that it completely liquefies the gelatine. The

Fig. 1$\alpha$

Fig. 2$\alpha$

Fig. 3$\alpha$

culture tubes present, therefore, a funnel-shaped area of liquefied gelatine, while the fungi themselves fall to the bottom of the funnel (see Fig. 2*a*).

Fig. 4.

This fungus forms furrows in the plates, and if the plate is turned on its edge the whole mass of fungus flows from one end of the furrow towards the other, or slides quite off the plate.

The $\delta$ fungus (Fig. 3) forms completely *opaque* masses which may have a slight yellowish tinge, provided the gelatine itself is yellowish. It has a small surface growth, and liquefies the gelatine only to a slight extent. In cultures on plates which are two or three days old, the row of fungus appears to lie in a trough, or depression in the gelatine. It does not move, however, when the plate is turned on edge (see Fig. 3*a*).

For the fungus of Fig. 4 I have not yet been able to establish definite peculiarities of growth. As far as my observations have at present extended, it differs from that of Fig. 3, in that it is almost entirely wanting in surface growth, and forms colorless masses, even in colored media. It does not liquefy the gelatine. Viewed by transmitted light it appears to have a bluish tinge, and a slight opalescence. It grows, however, very slowly, and I have consequently as yet been unable to establish certain and definite characteristics for it. The fungus described on page 227 grows still more slowly at gelatine temperature, and I cannot at present give any microscopical features by which cultures on gelatine may be distinguished.

The most important feature connected with all these fungi, especially the coccus forms, is that they possess a ferment activity ; in other words, they are capable of producing acid out of sugar, or, in the human mouth, out of starch, by the aid of the diastatic action of the saliva. They may consequently all be looked upon as factors in the decay of the teeth. I would not venture to say that the *a* fungus is more concerned in the process of caries than all the rest together; nevertheless, such is the constancy with which I

have found it, that if any one else should make the assertion I would have no reason for contradicting him. Cultivated in liquid substrata, none of them form films or skins upon the surface of the liquid, but powdery or fleecy precipitates upon the bottom and sides of the vessel. None, so far as I have observed, produce an evolution of carbonic acid in solutions containing sugar, nor do they appear to suffer when the access of oxygen is restricted.

A question of great importance, not only for dentists but for general physicians, and, in fact, for everybody, is that relating to the possible pathogenic nature of these fungi. We find in the works of Leyden and Jaffé, Haussmann, Bollinger, James Israel, etc., sufficient ground for the statement that "these fungi, in all parts of the human body which they reach, can play the same malignant role as upon the teeth." Gangrene of the lungs, abscesses of the mouth and throat, chronic pyæmia, etc., etc., have by various authors been ascribed to the action of the fungi of the human mouth. Raynaud, Lannelongue, and Pasteur produced what they called *maladie nouvelle* by inoculating rabbits with the saliva of a child bitten by a mad dog. And A. Fraenkel has in a number of cases produced sputum-septicæmia by inoculating rabbits with his own saliva.

We ask ourselves then the question: may not many of our obscure cases of infectious disease which now and then appear after extraction, or other dental operations, and which are, without further examination, attributed to the unclean instruments or hands of the dentist, be the result of an infection produced by microorganisms in the patient's own mouth? If a man's saliva contains organisms which, when brought into the blood of a rabbit, occasion death in twenty-four hours, would it be a matter of no consequence to produce so large a wound in his mouth as that caused by the extraction of a tooth? For the purpose, if possible, of throwing some light upon this question, I have undertaken a series of experiments for determining whether the organisms which are most commonly found in the human mouth possess the power of producing death (by septicæmia or otherwise) by inoculation. These experiments, as well as the others recorded in this article, I

have in fact only begun. My absence from home, however, prevents my carrying them on during the summer months, and I have determined, therefore, to present the results which I have already obtained, few and imperfect as they are.

The inoculations have thus far been performed on three rabbits, one rat, and six white mice. They were made partly with a mixture of the two fungi $\alpha$ and $\gamma$, and partly with saliva which had been kept in sterilized calf's broth for fifteen hours, at blood temperature.

Each rabbit received 1 c. c. of the infected liquid, injected directly into the lung or abdominal cavity; the rat 0,2 c. c., and the mice 0,1 c. c.

*Exp.* 1. Small rabbit inoculated with 1 c. c. in the abdominal cavity.

In the course of a few hours the rabbit appeared evidently ill; refused to eat, and remained quiet in the corner of the cage. In twenty-four hours diarrhœa appeared, with a slight elevation of temperature. These symptoms increased during the next day, till fifty hours after the time of inoculation it was found at the point of death. The examination showed the blood to be almost entirely free from organisms, and no indication of septicæmia. Living fungi were found, however, in the abdominal cavity, and a large part of the right lobe of the liver was completely riddled with masses of fungi; also in the fæces were found enormous numbers, which, morphologically, were identical with those in the liver, their entrance into the alimentary canal from the liver being easily accomplished. I unfortunately neglected, however, to establish their identity by the proper cultures.

*Exp.* 2. Rabbit inoculated as in Exp. 1.

The animal manifested a slight indisposition on the second day, from which it soon recovered.

*Exp.* 3. Rabbit inoculated in the right lung with saliva which had been kept in sterilized calf's broth for fifteen hours, at thirty-seven degrees Centigrade. No effect apparent.

*Exp.* 4. White rat, injection in abdominal cavity.

The animal remained well.

*Exps.* 5–11. Seven white mice; five inoculated in abdominal cavity with $\alpha$ and $\gamma$ fungi ; two in the lungs with saliva in calf's broth. Of the former two died at about the fortieth hour under the same symptoms as in Exp. 1. Great numbers of fungi were found in the abdominal cavity, which by culture on gelatine proved to be the $\gamma$ fungus. A number of colonies were likewise found in the liver. Microtome sections of the liver of the rabbit stained in Fuchsine show, when examined under the microscope with sufficient light to drown the tissue, a distribution of the fungi very similar to that often seen in the outermost layers of carious dentine. (See Fig. 5.) Of course, no definite conclusion can be drawn from a few experiments. They are, however, sufficient to show that these fungi certainly do possess a pathogenic character, and when brought into other parts of the human body may be able, under predisposing conditions, to produce disastrous results. Especially the continual swallowing of these fungi in great numbers may, by their ferment activity alone in the course of time, produce very serious derangements of the stomach and alimentary canal, since the small percentage of hydrochloric acid in the stomach, even in the presence of the normal quantity of pepsin, is not sufficient to devitalize them. It was with a certain degree of satisfaction that I have failed thus far to find the coccus of sputum-septicæmia in my own saliva. It is, however, very desirable that experiments should be made with the saliva of many persons, for the purpose, if possible, of determining in what proportion of cases this fungus is present.

Fig. 5

Messrs. Underwood and Milles have endeavored to repeat some of my earliest experiments in the production of artificial caries, but under those very abnormal conditions against which I entered warning in the INDEPENDENT PRACTITIONER, page 229. Failure was the necessary result. They performed, further, a very elaborate experiment, lasting six months, in which the baths became so putrid and offensive that "they quit the experiment with relief." They naturally produced no caries, thereby furnishing an admirable confirmation of the fact to which I have so often called attention, that it is impossible to produce even a trace of caries by putrefaction alone. They tried a third experiment, putting the fungi under such abnormal conditions that they could not produce acid, and of course failed again, once more confirming the fact that I have long since established, that we can have no caries without acid. With these experiments they risk the statement that artificial caries is probably an impossibility. The production of artificial caries is a *fait accompli*, and to deny its possibility is only to endanger the reputation of him who denies.

They state further that they can find no softened dentine which does not contain micro-organisms. This, however, is contrary to the experience of a great many American microscopists, and, moreover, as I have elsewhere stated, I shall take with me to the next meeting of the American Dental Society of Europe several hundreds of specimens of carious dentine, and be ready to show the areas of softened, non-infected dentine, on any one or on all of them.

Messrs. Underwood and Milles understand me, in the third place, as being of the opinion that all the micro-organisms connected with caries of the teeth are only different forms of one fungus. The readers of the INDEPENDENT PRACTITIONER know better. I have stated simply that *one* of the many fungi found in the human mouth in connection with caries of the teeth, may produce different forms of development. This is the fungus which I have designated by the prefix $\beta$. It is scarcely necessary to add that I am always prepared to prove its existence microscopically, as well as on the authority of many of the best mycologists of Germany.

No one, I think, will deny that within the last few years I have done a large amount of work, and contributed some evidence towards the solution of the problem of dental caries. The amount of material dealt with, and the ground gone over, have been so extent sive that it has been absolutely impossible, with the greatest efforts, to remain as long by each step as would have been desirable. It may be, therefore, that at some points the subject has not been presented with sufficient clearness or decisiveness; it may be, too, that at some points the conclusions have been faulty, since I make no pretension to infallibility. Time will show whether this is the case. At present I know of no important change which I could make, if I were to re-write all my contributions of the last three years.

I desire to give, in closing, a very short resumé of the work which I have accomplished.

1. I convinced myself by the examination of some thousands of slides of carious dentine, that micro-organisms were always present, and that they, without any doubt, were the cause of various anatomical changes which were found to take place in the structure of the dentine during caries. (Here, of course, the question of priority does not suggest itself; Leber and Rottenstein, as is well known, were the first to give definite expression to this fact.)

2. I proved, at the same time, that the invasion of the micro-organisms was not, in the majority of cases, simultaneous with the softening of the dentine, but that large areas of softened dentine could be found that contained no fungi. Of all those who examined my preparations in America, no one, whatever his theory, ever once denied this fact. I concluded from this that the softening of the dentine went in advance of the invasion of the organisms.

3. I determined by analyses of masses of carious dentine, sufficiently large to give reliable results, that the softening of the dentine is of the nature of a true decalcification. That the decalcification of the outer layers is almost complete, and diminishes in degree as we advance towards the normal dentine. Furthermore, that the same relations maintain in dentine softened in a mixture of saliva and bread, or in weak organic acids; also, that in a mass of carious dentine the lime-salts had been removed to a much greater extent than the organic matter.

4. I maintained from the first that the softening of the dentine was produced by acids, for the most part generated in the mouth by fermentation. I had, however, no direct proof of this.

5. I *proved* that fungi exist in great numbers in the human saliva and in carious dentine, which have the power to produce acid under conditions which are constantly present in the human mouth. I determined this acid, for one of the fungi, at least, to be the ordinary ferment, lactic acid.

6. I produced caries artificially, which under the microscope cannot be distinguished from natural caries, by subjecting sound dentine to the action of these fungi in fermentable solutions.

7. I determined the influence of various antiseptics and filling materials upon the fungi of caries.

8. I isolated various forms of these fungi, and determined *in part* the conditions most favorable to their development, their characteristic reaction upon gelatine, their physiological action, their effect when inoculated into the system of lower animals, and their possible connection with certain obscure diseases generally attributed to the carelessness of the dentist.

My continual search has been after facts, and such facts as I have obtained I have presented before the profession, never putting before them either theory or speculation, nor anything which was not the result of severe and continued labor, and in this spirit I propose to prosecute this work, as well as any other that I may undertake in the interest of the profession.

BERLIN, May 21, 1884.

NOTE.—Since writing the above, I have succeeded in producing death by septicæmia of both mice and rabbits, by injecting into the lung saliva from the mouth of a perfectly healthy person.

## Figures and Tables

**Fig. 1 f1:**
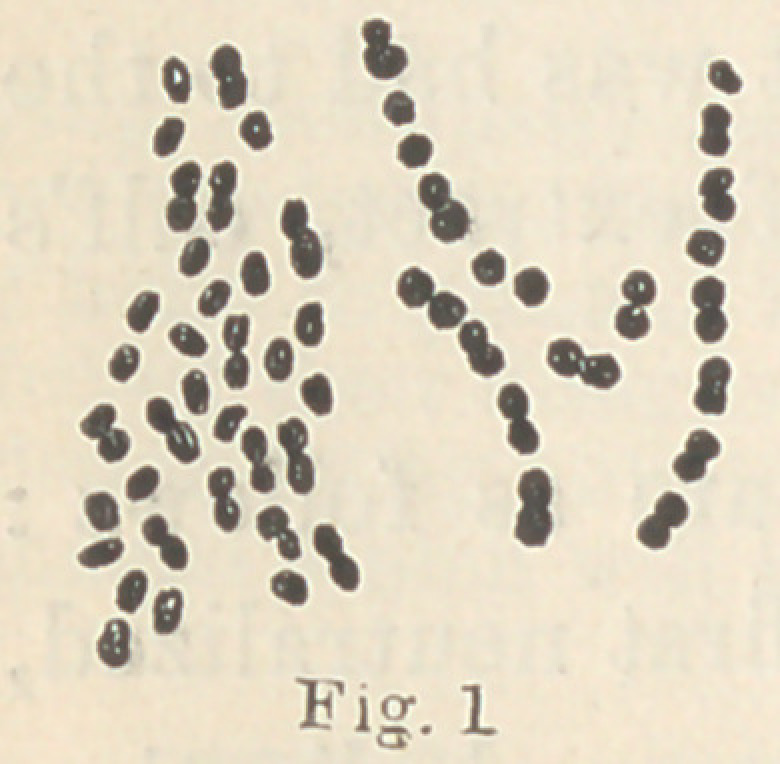


**Fig. 2 f2:**
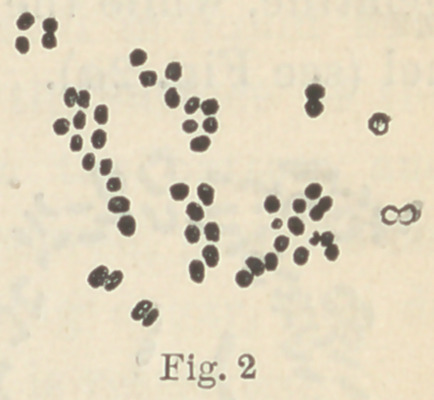


**Fig. 3 f3:**
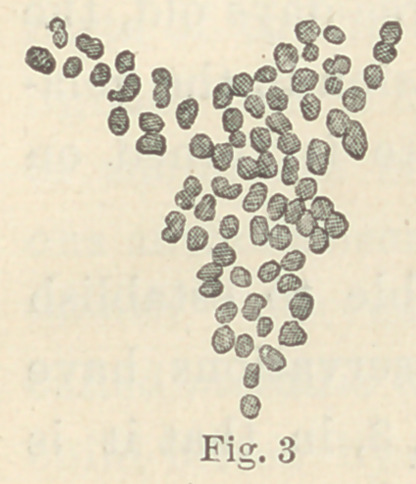


**Fig. 1a f4:**
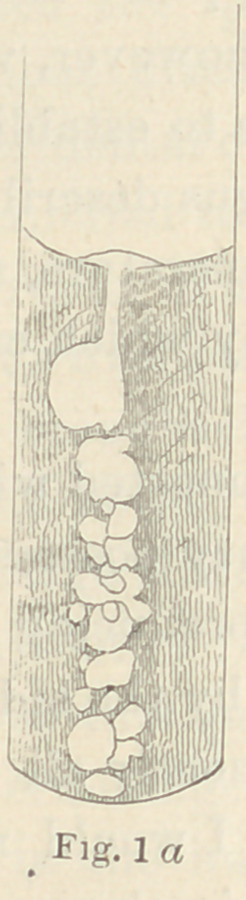


**Fig. 2a f5:**
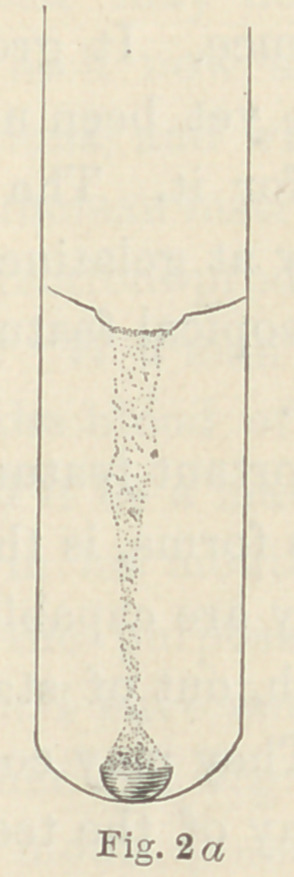


**Fig. 3a f6:**
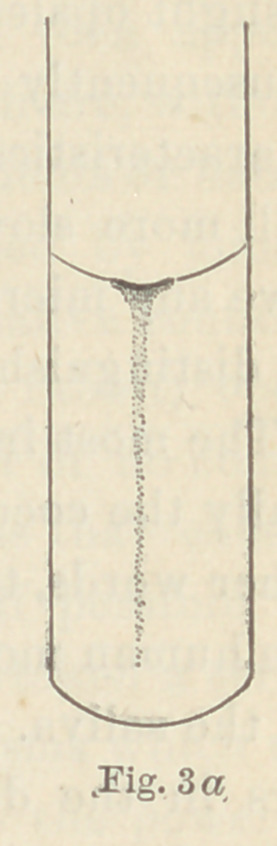


**Fig. 4 f7:**
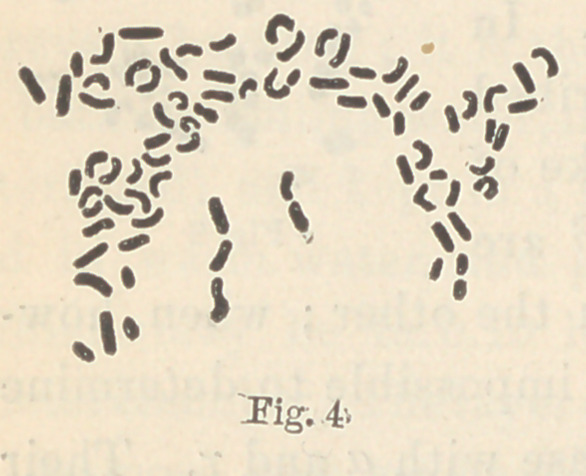


**Fig. 5 f8:**